# Using Fenton Oxidation to Simultaneously Remove Different Estrogens from Cow Manure

**DOI:** 10.3390/ijerph13090917

**Published:** 2016-09-15

**Authors:** Minxia Sun, Defu Xu, Yuefei Ji, Juan Liu, Wanting Ling, Shunyao Li, Mindong Chen

**Affiliations:** 1 Institute of Organic Contaminant Control and Soil Remediation, College of Resources and Environmental Sciences, Nanjing Agricultural University, Nanjing 210095, China; 2013103044@njau.edu.cn (M.S.); yuefeiji@njau.edu.cn (Y.J.); liujuan@njau.edu.cn (J.L.); 2015103034@njau.edu.cn (S.L.); 2 Jiangsu Key Laboratory of Atmospheric Environment Monitoring and Pollution Control, School of Environmental Science and Engineering, Nanjing University of Information Science & Technology, Nanjing 210044, China; defuxu1@163.com

**Keywords:** estrogen, Fenton oxidation, cow manure, reaction condition, estriol, bisphenol A, diethylstilbestrol, estradiol, ethinyl estradiol

## Abstract

The presence of estrogens in livestock excrement has raised concerns about their potential negative influence on animals and the overall food cycle. This is the first investigation to simultaneously remove estrogens, including estriol (E3), bisphenol A (BPA), diethylstilbestrol (DES), estradiol (E2), and ethinyl estradiol (EE2), from cow manure using a Fenton oxidation technique. Based on the residual concentrations and removal efficiency of estrogens, the Fenton oxidation reaction conditions were optimized as follows: a H_2_O_2_ dosage of 2.56 mmol/g, a Fe(II) to H_2_O_2_ molar ratio of 0.125 M/M, a solid to water mass ratio of 2 g/mL, an initial pH of 3, and a reaction time of 24 h. Under these conditions, the simultaneous removal efficiencies of E3, BPA, DES, E2, and EE2, with initial concentrations in cow manure of 97.40, 96.54, 100.22, 95.01, and 72.49 mg/kg, were 84.9%, 99.5%, 99.1%, 97.8%, and 84.5%, respectively. We clarified the possible Fenton oxidation reaction mechanisms that governed the degradation of estrogens. We concluded that Fenton oxidation technique could be effective for efficient removal of estrogens in livestock excrement. Results are of great importance for cow manure reuse in agricultural management, and can be used to reduce the threat of environmental estrogens to human health and ecological safety.

## 1. Introduction

Endocrine disrupting chemicals (EDCs) are chemical substances that can cause adverse health effects on organisms, or cause changes in the endocrine function of an organism’s offspring. EDCs are divided into natural estrogens, synthetic estrogens, phytoestrogens, and various industrial chemicals (e.g., pesticides, persistent organochlorines, organohalogens, alkyl phenols, heavy metals) [1]. Environmental estrogen contamination has become the third largest environment problem after ozone depletion and global warming, and have been described as a time bomb threatening human survival [2]. Animals and humans can be exposed to environmental estrogens through different pathways, such as via the digestive tract, respiratory tract, and skin contact, while long-term retention can occur in animal and human body adipose tissue. When the accumulation of estrogens in the body reaches a certain concentration, it is released from fat tissue into the blood stream, and adverse effects on organisms can result [3]. Some natural estrogens, such as estriol (E3) and estradiol (E2), and synthetic estrogens, such as ethinyl estradiol (EE2) and diethylstilbestrol (DES) have previously been found to be estrogenic and are regarded as the most potent ECDs in the environment [4].

Intensive farming is a major source of environmental estrogens, which stem mainly from the drainage of the normal physiological secretions from livestock and poultry animals, and the addition of growth hormones (assimilation hormones) in feed. Through the agricultural application of animal manures, estrogens can enter the soil and water, and become a potential threat to the environment. Zheng et al. detected estrogen concentrations up to 2103 ± 123 μg/kg from fresh cow manure in typical cow farms in the United States [5]. Natural steroid estrogens are commonly found at high concentrations in farm manure. Through the application of farm manure, estrogens can enter the farmland environment, and subsequently enter groundwater and river water via farmland irrigation, surface runoff, and infiltration (macropore flow). Tashiro et al. determined that all main and tributary rivers within a selected river basin were polluted by estrogen [6]. Peterson et al. found that in aquifers deep underground in the Kashgar region the 17β-E2 levels were as high as 6–66 ng/L because of poultry and animal feces pollution [7]. In China, every year nearly four billion tons of livestock and poultry dung are applied to the soil and enter the environment. Because of the use of growth promoting agents in modern farming and the physiological activities of livestock and poultry, large volumes of estrogens are discharged into livestock and poultry dung and enter the soil and water environment through the agricultural use of animal feces, potentially polluting farmland soil, surface water, and groundwater; interfering with the normal endocrine function of aquatic organisms; threatening the ecological environment; and eventually causing harm to human beings. However, details of how to remove the estrogen in livestock and poultry feces are not readily available.

The degradation of environmental estrogens is given priority over microbial biodegradation and photolysis. Vader et al. reported that nitrifying activated sludge could degrade EE2 [8]. The experiment showed that nitrification and microbial degradation processes used for EE2 removal were related to the microbial metabolism, and that full biodegradation of EE2 took six days. Activated sludge degradation led to the disappearance of its estrogenic activity. Tanaka et al. studied the degradation of five endocrine disruptors in bottom sediments [9]. The experiment showed that bisphenol A (BPA) could be removed by extracellular polymerization and degradation by basidiomycete laccase. Biodegradation is an important degradation pathway for environmental estrogens, although the time required for estrogen biodegradation is long and the process relatively inefficient. Segmuller et al. identified natural oxidation and light degradation (using xenon lamps as a light source) products of EE2 in solution [10]. Coleman et al. studied the catalytic photodegradation (TiO_2_ catalyst) of EE2 [11]. The experiment showed that the micro molar concentration (0.05–3 μmol/L) EE2 solution was degraded by 98% in 3.5 h. Yoshida et al. considered that although TiO_2_ was important in the catalytic degradation of pollutants, it could not be applied in practical wastewater treatment because impurities would reduce the UV light degradation efficiency [12].

Advanced oxidation processes (AOPs) have received particular attention for the degradation of emerging micropollutants such as EDCs and pharmaceuticals [13]. In various AOPs, hydroxyl radicals (HO^·^) are produced to oxidize organic pollutants either completely into carbon dioxide, water, and inorganic salts [14] or incompletely into less hazardous intermediates [15]. The HO^·^ radical is highly oxidative and can react with most organic pollutants, with a 10^9^ M^−1^·s^−1^ second-order rate constant. Therefore, HO^·^ can non-selectively oxidize many organic and inorganic contaminants. AOPs have emerged as important technologies for eliminating estrogens in the environment. 

The Fenton process (Fe(II)/H_2_O_2_), one of the most promising AOPs, is highly efficient for mineralization and can be performed at ambient temperature. As an oxidation technique, the Fenton process has been widely used to remove many refractory pollutants in previous studies, such as 2,6-dimethylanline [16], polychlorinated biphenyls (PCBs) [17], 2,4,6-trinitrotoluene (TNT) [18], pharmaceutical wastewater [19], dye wastewater [20], and landfill leachate [21]. However, the available literature regarding the oxidation degradation of estrogen by the Fenton process is limited, with most studies involving a solution system for a single estrogen. No data are available regarding the removal of several estrogens in livestock and poultry waste by the Fenton process. 

To this end, our aim was to investigate Fenton oxidation of E3, BPA, DES, E2, and EE2 in cow manure. We examined the effects of H_2_O_2_ dosage, Fe(II) to H_2_O_2_ molar ratio, solid to water mass ratio, initial pH value, and reaction time on estrogen removal efficiency. Results of this work will provide a useful and convenient technique suitable for removing estrogens from livestock and poultry. 

## 2. Materials and Methods

### 2.1. Chemicals

Table 1 shows the general properties of the estrogens we investigated. Standards of E3, BPA, DES, E2, and EE2 (purity > 98%) were purchased from Sigma-Aldrich (St. Louis, MO, USA). High-performance liquid chromatography (HPLC) methyl cyanide and methanol were used as organic solvents and obtained from Sigma-Aldrich (St. Louis, MO, USA). Other principal chemicals were purchased as analytical grade and used without further purification. Stock estrogen solutions (2.5 g/L) of each estrogen were prepared in acetone in glass tubes and stored at 4 °C.

### 2.2. Cow Manure

Cow manure was obtained from a farm at Nanjing, and contained 14.5% organic matter, 0.30%–0.45% nitrogen, 0.15%–0.25% phosphorus, and 0.10%–0.15% potassium. The initial pH value of cow manure was 7.1 ± 0.11. 

#### 2.2.1. Cow Manure Moisture Content

We used the cow manure moisture content to calculate the hydrogen peroxide content. A 5 g sample of cow manure was placed in an aluminum box and dried for 2 h at 110 °C. The cow manure moisture content was calculated as follows:

*S_M_* (%) = (*M*_1_ − *M*_2_)/*M*_1_ × 100 (1)

where *S_M_* is the cow manure moisture content, *M*_1_ is the weight of the cow manure before drying, and *M*_2_ is the weight of the cow manure after drying. The calculated moisture content of the cow manure used in the experiment was 1.17%. 

#### 2.2.2. Preparation of Estrogen-Contaminated Cow Manure

Cow manure was freeze-dried, sieved to 0.83 mm, and contaminated with a mixture of five estrogens using the following procedure. Certain amounts of estrogens were dissolved in acetone and mixed with 200 g of cow manure. Acetone was then evaporated and the contaminated cow manure was mixed with 800 g of uncontaminated cow manure. The cow manure was then fully homogenized. The initial contents of E3, BPA, DES, E2, and EE2 detected in cow manure were 97.40 ± 3.55, 96.54 ± 0.42, 100.22 ± 0.35, 95.01 ± 1.01, and 72.49 ± 1.55 mg/kg respectively. 

### 2.3. Fenton Oxidation Experiments

We conducted Fenton oxidation experiments in a 30-mL glass centrifuge tube at 25 °C. A 1.0 g sample of cow manure containing estrogens was transferred to the centrifuge tube, and distilled water was added. The initial pH value of the sludge was adjusted to 3.0–11.0 using NaOH and HCl as appropriate. Specific amounts of ferrous sulfate (1 mol/L) and H_2_O_2_ (30% *v*/*v*) were immediately added. The reaction was allowed to proceed for a specific length of time. Then 2 mol/L NaOH was injected into the centrifuge tube to increase the pH to 10 to terminate the reaction. The cow manure was then separated by centrifugation and freeze-dried for estrogen analysis. 

### 2.4. Estrogen Analysis in Cow Manure

To determine the E3, BPA, DES, E2, and EE2 concentrations, we added 15 mL of ethyl acetate extraction agent to the freeze-dried cow manure. Tubes were closed with a Teflon-liner cap, and cow manure was extracted by ultrasonication for 1 h. Samples were centrifuged for 10 min at 4000 rpm to separate cow manure from aqueous solution and 5 mL of supernatant was dehydrated by percolation through a column with Na_2_SO_4_ anhydride. The column was then eluted with 10 mL 1:1 (volume ratio) of ethyl acetate and methanol. Elutions were collected and blown dry with nitrogen (N_2_), and the extract was then resuspended in methanol to a final volume of 2 mL. After filtration through a 0.22-μm filter, estrogens were detected by HPLC.

Estrogens were analyzed with a HPLC system fitted with an ultraviolet detector (UV) and an Intertsil ODS-SP-C_18_ column (5 μm, 150 × 4.6 mm), using methanol (25%)/acetonitrile (30%)/water (45%) as the mobile phase at a flow rate of 1 mL/min. Chromatography was performed at 40 °C. E3, BPA, DES, E2, and EE2 were detected at 280, 230, 230, 280, and 280 nm, respectively.

The removal efficiency of estrogens in cow manure by Fenton oxidation was calculated as follows:

Removal efficiency (%) = (*C*_0_ − *C*_t_)/*C*_0_ × 100 (2)

where *C_t_* and *C_0_* are the estrogen concentrations in cow manure at times *t* and *0*, respectively. 

### 2.5. Statistical Analysis

All data were processed with Microsoft Office Excel and SPSS version 13.0 (SPSS Inc., Chicago, IL, USA). Every data point in the figures is an average value. The standard deviations (SDs), obtained from three parallel samples, are shown in the figures as error bars. Data were analyzed using one-way ANOVA at α = 0.05. 

## 3. Results

### 3.1. H_2_O_2_ Dosage

**Figure 1:**
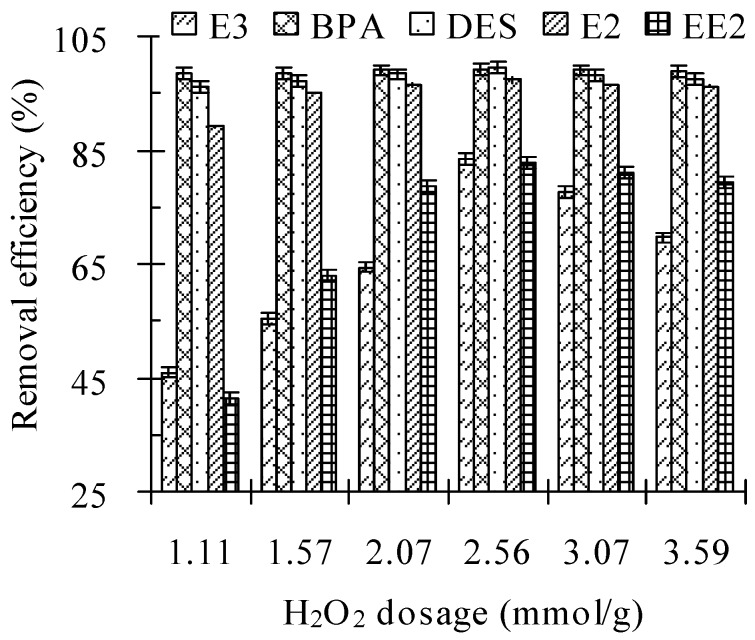
Effect of H_2_O_2_ dosage on the removal efficiency of estrogens from cow manure by the Fenton oxidation process. Note: the Fe(II) to H_2_O_2_ molar ratio was 0.10 M/M; the solid to water mass ratio was 2 g/mL; the initial pH value was 3.0; the reaction time was 24 h. Error bars represent standard deviations.

The H_2_O_2_ dosage significantly influenced the removal of tested estrogens by Fenton oxidation in cow manure. As shown in Table 2 and Figure 1, the residual concentrations of estrogens in cow manure after Fenton oxidation for 24 h dramatically decreased when the H_2_O_2_ dosage increased from 0 to 2.56 mmol/g, and then increased with H_2_O_2_ dosage from 2.56 to 3.59 mmol/g. As a consequence, the removal efficiencies of estrogens were first enlarged and then reduced when the H_2_O_2_ dosage increased from 0 to 2.56 mmol/g and then to 3.59 mmol/g. The lowest concentrations of E3, BPA, DES, E2, and EE2 were observed at H_2_O_2_ dosage of 2.56 mmol/g, and were 15.92, 0.29, 0.48, 2.14 and 12.11 mg/kg with corresponding removal efficiencies of 83.4%, 99.3%, 99.5%, 97.3%, and 82.9%, respectively. At the test H_2_O_2_ dosage of 1.11 to 3.59 mmol/g, the respective removal efficiencies of E3, BPA, DES, E2, and EE2 were always higher than 46.0%, 98.37%, 96.14%, 89.15%, and 41.28%, respectively. However, the removal efficiencies of BPA, DES, and E2 by Fenton oxidation with a H_2_O_2_ dosage of 1.11–3.59 mmol/g were much higher than those of E3 and EE2. 

### 3.2. Fe(II) to H_2_O_2_ Molar Ratio

**Figure 2:**
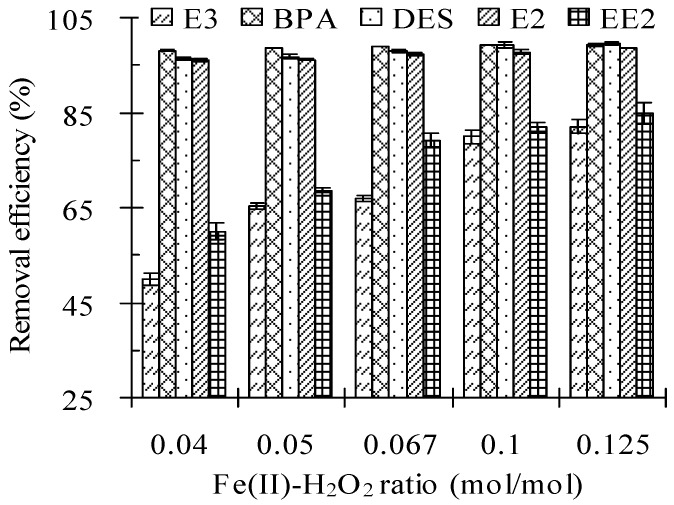
Effect of the Fe(II) to H_2_O_2_ molar ratio on the removal efficiency of estrogens from cow manure by the Fenton oxidation process. Note: the H_2_O_2_ dosage was 2.56 mmol/g; the solid to water mass ratio was 2 g/mL; the initial pH value was 3.0; the reaction time was 24 h. Error bars represent standard deviations.

A larger Fe(II) to H_2_O_2_ molar ratio improved the removal of the five tested estrogens in cow manure by the Fenton oxidation technique, as shown in Table 3 and Figure 2. The residual concentrations of estrogens clearly decreased and their removal efficiency was enhanced when the Fe(II) to H_2_O_2_ molar ratio increased from 0 to 0.125 M/M in the test system. The rate of removal of target compounds increased when the molar ratio increased from 0.04 to 0.125 M/M. When the Fe(II) to H_2_O_2_ molar ratio increased from 0.04 to 0.125 M/M, the removal efficiencies of E3, BPA, DES, E2, and EE2 increased from 50.0%, 98.1%, 96.4%, 96.0%, and 60.0% to 82.1%, 99.3%, 99.5%, 98.6%, and 84.9%, respectively. The removal ratios of BPA, DES, and E2 were always higher than 96% at the test Fe(II) to H_2_O_2_ molar ratio of 0.04–0.125 M/M, and were much higher than the removal ratios for E3 and EE2. 

### 3.3. Solid to Water Mass Ratio

**Figure 3:**
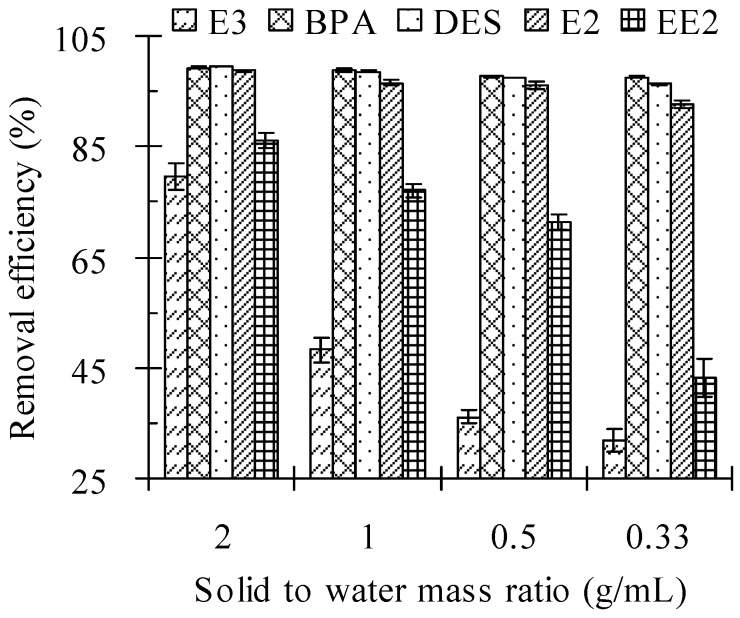
Effect of the solid to water mass ratio on the removal efficiency of estrogens from cow manure by the Fenton oxidation process. Note: Fe(II) to H_2_O_2_ molar ratio = 0.10 M/M; H_2_O_2_ dosage = 2.56 mmol/g; initial pH values = 3.0; reaction time = 24 h. Error bars are standard deviations.

The effect of the solid (cow manure) to water mass ratio on the removal of the five tested estrogens by Fenton oxidation is given in Table 4 and Figure 3. A smaller solid to water mass ratio resulted in a lower residual concentration and higher removal efficiency of test estrogens in cow manure. The residual concentrations of E3, BPA, DES, E2, and EE2 in cow manure after the Fenton oxidation process increased from 19.56, 0.38, 0.54, 0.81, and 11.12 mg/kg to 65.66, 2.05, 3.58, 4.54, and 41.84 mg/kg, respectively, with a decrease in the solid to water mass ratio from 2 to 0.33. The corresponding removal efficiency of E3, BPA, DES, E2, and EE2 decreased from 79.47%, 99.23%, 99.41%, 98.74%, and 85.97% to 31.92%, 97.52%, 96.32%, 92.59%, and 43.28%, respectively. E3 and EE2 are more difficult to oxidize than BPA, DES, and E2, with much lower removal efficiencies at the tested solid to water mass ratio.

### 3.4. Initial pH Values

**Figure 4:**
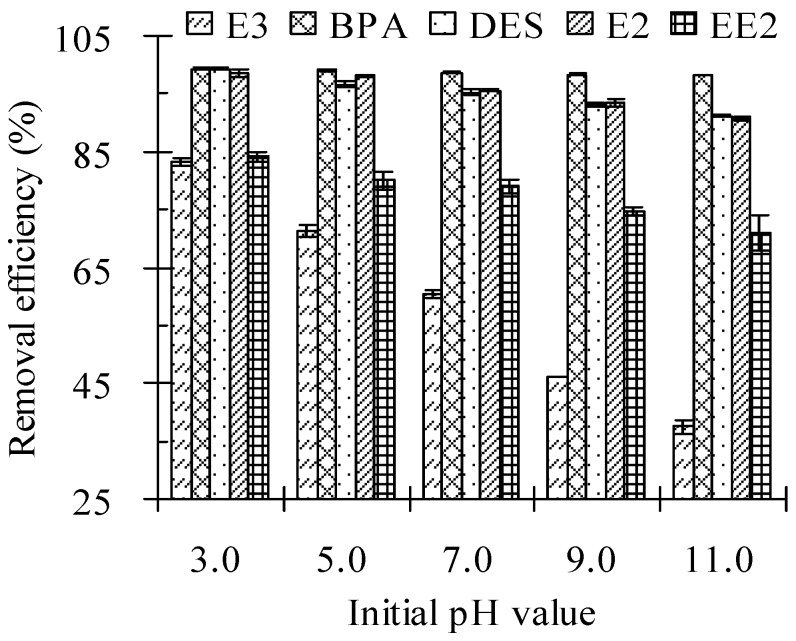
Effect of initial pH values on the removal efficiency of estrogens from cow manure by the Fenton oxidation process. Note: Fe(II) to H_2_O_2_ molar ratio = 0.10 M/M; H_2_O_2_ dosage = 2.56 mmol/g; solid to water mass ratio = 2 g/mL; reaction time = 24 h. Error bars are standard deviations.

The initial pH values of the cow manure-water sludge significantly influenced the removal of the five tested estrogens by the Fenton oxidation process. As displayed in Table 5 and Figure 4, the residual concentrations of estrogens in cow manure were clearly enhanced, while the removal efficiency decreased as the initial pH values increased from 3 to 11.0, because pH can influence the generation of the HO· radical. When pH values decreased from 11.0 to 3.0, the removal efficiencies of E3, BPA, DES, E2, and EE2 increased from 37.4%, 98.2%, 91.1%, 90.8%, and 70.9% to 83.2%, 99.4%, 99.3%, 98.5%, and 84.1%, respectively. For BPA, DES, and E2, their removal efficiencies at test pH values were always >90%, while the removal efficiencies of E3 and EE2 were much smaller (always <84.08%). 

### 3.5. Reaction Time

**Figure 5:**
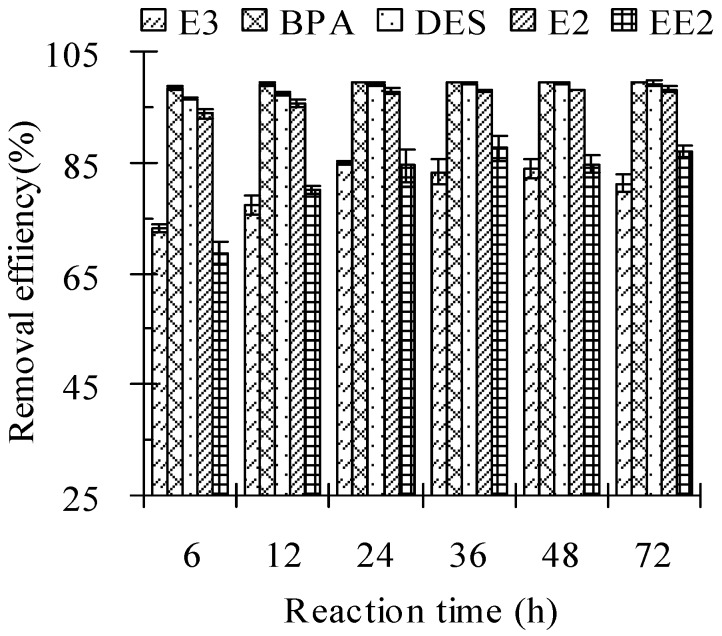
Effect of reaction time on the removal efficiency of estrogens from cow manure by the Fenton oxidation process. Note: the Fe(II) to H_2_O_2_ molar ratio was 0.10 M/M; the H_2_O_2_ dosage was 2.56 mmol/g; the solid to water mass ratio was 2 g/mL; the initial pH value was 3.0. Error bars represent standard deviations.

The removal of the five tested estrogens in cow manure by Fenton oxidation was very fast during the first 6 h of the reaction (Table 6 and Figure 5). Over the 6 h period, 73.15%, 98.43%, 96.48%, 93.79%, and 68.61% of E3, BPA, DES, E2, and EE2 in cow manure was degraded. Extending the reaction time from 6 to 24 h led to lower residual concentrations and a higher removal efficiency of the test estrogens in cow manure. The removal efficiencies were 84.9%, 99.5%, 99.1%, 97.8%, and 84.5% for E3, BPA, DES, E2, and EE2, respectively, for a reaction time of 24 h. However, when the reaction time was prolonged from 24 to 72 h, the residual concentrations and removal efficiencies of the estrogens did not change significantly (Table 6 and Figure 5). As a consequence, 24 h is the optimum reaction time for the removal of test estrogens in cow manure by the Fenton oxidation technique. 

## 4. Discussion

To the best of our knowledge, this is the first investigation of the removal of different estrogens in cow manure by a Fenton oxidation technique. We systematically investigated the effects of H_2_O_2_ dosage, Fe(II) to H_2_O_2_ molar ratio, solid to water mass ratio, initial pH value, and reaction time. Based on the residual concentrations and removal efficiency of the five tested estrogens using Fenton oxidation, we recommend a H_2_O_2_ dosage of 2.56 mmol/g; a Fe(II) to H_2_O_2_ molar ratio of 0.125 M/M; a solid to water mass ratio of 2 g/mL; and an initial pH value of 3. The extension of the reaction time may increase the cost of construction. Taking both estrogen removal and cow manure treatment costs into consideration, we recommend a reaction time of 24 h. 

As such, the reaction conditions of the Fenton oxidation were optimized as follows: a H_2_O_2_ dosage of 2.56 mmol/g, a Fe(II) to H_2_O_2_ molar ratio of 0.125 M/M, a solid to water mass ratio of 2 g/mL, an initial pH of 3, and a reaction time of 24 h. Under these conditions, the simultaneous removal efficiencies of E3, BPA, DES, E2, and EE2, with initial concentrations in cow manure of 97.40, 96.54, 100.22, 95.01, and 72.49 mg/kg, were 84.9%, 99.5%, 99.1%, 97.8%, and 84.5%, respectively. 

With Fe^2+^ as a catalyst, H_2_O_2_ decomposed to generate hydroxyl free radicals (HO^·^) and hydroxyl ions (OH^−^) under acid conditions. In the middle of the oxidation process a continuous chain reaction occurred, in which the generation of HO^·^ initiated the chain and the formation of other free radicals and reaction intermediates occurred as chain nodes. Between the various free radical reactions or free radical interactions with other substances free radicals were consumed, which terminated the chain reaction [22,23,24]. The reaction mechanism was as follows: 

Chain initiation:

Fe^2+^ + H_2_O_2_ → Fe^3+^ + HO^·^ + OH^−^ k_1_ ≈ 70 M^−1^·s^−1^ (3)

Chain propagation:

Fe^3+^ + H_2_O_2_ → Fe^2+^ + ·HO_2_ + H^+^ k_2_ = 0.01 M^−1^·s^−1^ (4)

Fe^3+^ + ·HO_2_ → Fe^2+^ + O_2_ + H^+^ k_3_ = 1.2 × 10^6^ M^−1^·s^−1^ (at pH = 3) (5)

HO^·^ + H_2_O_2_ → H_2_O + ·HO_2_ k_4_ = 3.3 × 10^7^ M^−1^·s^−1^ (6)

Chain termination:

Fe^2+^ + HO^·^ → Fe^3+^ + OH^−^ k_5_ ≈ 3.2 × 10^8^ M^−1^·s^−1^ (7)

Fe^2+^ + ·HO_2_ → Fe^3+^ + HO_2_^−^ k_6_ = 1.3 × 10^6^ M^−1^·s^−1^ (at pH = 3) (8)

HO^·^ + HO^·^ → H_2_O_2_ (9)

HO^·^ + ·HO_2_ → O_2_ + H_2_O (10)

The above equations showed that H_2_O_2_ was both an initiator (Equation (3)) and quencher (Equation (6)) of HO^·^. The resulting HO^·^ could either add to C=C double bonds or abstract a H atom from the organic pollutants (RH) to generate an organic free radical (R^·^). The R^·^ could then be further oxidized or transferred, and eventually mineralized into carbon dioxide and water:

RH + HO^·^ → R^·^ + H_2_O → further oxidation (11)

In the Fenton process, organic pollutants compete with HO^·^ through Equations (6) and (7) for oxidation removal. The production of HO^·^ was key to the removal efficiency. Therefore, many factors that influence the Fenton reaction should be considered, such as the Fe(II) to H_2_O_2_ molar ratio, the H_2_O_2_ dosage, the solid to water mass ratio, the initial pH values, and the reaction time to determine the optimal reaction parameters, in order to improve the removal efficiency of organic pollutants.

We studied the reaction system of Fenton oxidation. The removal efficiencies of the five test estrogens increased when the H_2_O_2_ dosage increased from 1.11 to 2.56 mmol/g. However, as the H_2_O_2_ dosage further increased to 3.59 mmol/g, the removal efficiencies decreased. More HO^·^ radicals were produced at increased dosages of H_2_O_2_, resulting in a higher removal of the test estrogens. However, due to the HO^·^ being quenched by the excessive levels of H_2_O_2_, extremely high dosages of H_2_O_2_ did not improve the removal efficiency (Equation (6)).

The removal efficiencies of the tested estrogens increased when the molar ratio increased from 0.04 to 0.125 M/M. It is likely that more HO· radicals were generated at high molar ratios, but HO^·^ radicals were also scavenged by reacting with excess Fe(II) as shown in Equation (7) [25]. Excess HO^·^ radicals may have reacted with themselves, as shown in Equation (9) [26].

2H_2_O_2_ → O_2_ + 2H_2_O (12)

The removal efficiencies of the target estrogens decreased when the solid to water mass ratio decreased from 2 to 0.33 g/mL. When the solid to water mass ratio was 2 g/mL, the cow manure samples were fully mixed under the shock produced by a vortex mixing apparatus. With the increasing solid to water mass ratio, the contact area between reagent and sample was reduced [27], resulting in decreased removal efficiencies. 

The removal efficiencies of target estrogens increased when the initial pH values decreased from 11.0 to 3.0. The generation of HO^·^ radicals can be influenced by pH. A rise in pH can cause the formation of an inactive hydroperoxide anion (HO_2_^−^) due to H_2_O_2_ dissociation [28]. The HO_2_^−^ acts as an efficient scavenger of HO^·^ radicals as shown in Equation (13), which leads to the inhibition of degradation [24]. When the pH value was >4, Fe(II) was quickly oxidized to Fe(III) and formed an Fe(OH)_3_ precipitate. The concentration of free iron species decreased due to the formation of complexes that could not easily react with H_2_O_2_ [29]. The H_2_O_2_ became unstable and decomposed to oxygen when the pH was high. It also lost its oxidation ability, as shown in Equation (12) [30].

HO^·^ + HO_2_^−^ → H_2_O + ·O_2_^−^ (13)

The removal efficiency of the tested estrogens increased at longer reaction times. The degradation of estrogens was fast during the first 6 h but did not vary significantly when the reaction time was extended from 24 to 72 h. Reaction time was mainly influenced by the rate of HO^·^ formation and its reaction rate with organics [24]. At the beginning of the reaction, the concentration of HO· was high, which allowed considerable contact with the target estrogens. As HO^·^ was consumed, the degradation rates began to slow. 

An optimum value of H_2_O_2_ dosage was observed at about 2.56 mmol/g with the highest removal efficiency of tested estrogens from cow manure, and higher or lower amounts of H_2_O_2_ dosage reduced the removal efficiency. Other researchers reported the oxidation of estrogens in solution [31]. Frontistis et al. documented that in Fenton processes, the H_2_O_2_ to substrate concentration ratio at a constant catalyst concentration is a critical parameter to safeguard that the reaction is not limited by the quantity of hydroxyl radicals generated (i.e., at very low ratios) or it is not hindered by auto-scavenging reactions (i.e., at excessive ratios) [32]. In this investigation, the excess of H_2_O_2_ may act as scavenger for the OH radicals. 

## 5. Conclusions

This is the first investigation into the removal of different estrogens from cow manure by Fenton oxidation techniques. The reaction conditions of the Fenton oxidation were optimized to ensure the high removal efficiency of estrogens from cow manure as follows: the H_2_O_2_ dosage was 2.56 mmol/g, the Fe (II) to H_2_O_2_ molar ratio was 0.125 M/M, the solid to water mass ratio was 2 g/mL, the initial pH was 3, and the reaction time was 24 h. These results show that Fenton oxidation, as a new type of oxidation technology, would be effective and convenient for the simultaneous removal of highly toxic estrogens from cow manure. The results are of great importance for the reuse of cow manure in agricultural management, and will be valuable for reducing the threats to human health and ecological safety posed by environmental estrogens. 

## Figures and Tables

**Table 1:** General properties of the tested estrogens.

Compound	E3	BPA	DES	E2	EE2
Molecular structure	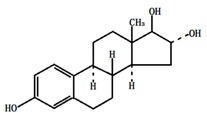	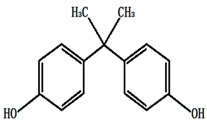	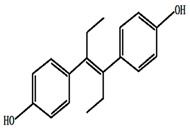	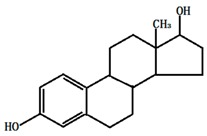	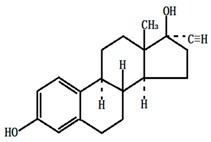
Molecular formula	C_18_H_24_O_3_	C_15_H_16_O_2_	C_18_H_20_O_2_	C_18_H_24_O_2_	C_20_H_24_O_2_
Molar mass (g/M)	288.4	228.3	268.36	272.38	296.4
Melting point (°C)	280~282	176~180	170~172	178~179	182~183
Solubility in water (mg/L)	13	13	10	1.51 ± 0.04	9.2 ± 0.09
p*K*_a_	10.4	10.7	10.3	10.5	11.3
log*K*_ow_	2.6	3.94	5.07	3.1	3.9

**Table 2:** Effect of H_2_O_2_ dosage on the residual concentrations of estrogens in cow manure by the Fenton oxidation process. Note: the Fe(II) to H_2_O_2_ molar ratio was 0.10 M/M; the solid to water mass ratio was 2 g/mL; the initial pH value was 3.0; the reaction time was 24 h.

H_2_O_2_ Dosage/(mmol/g)	The Residual Concentrations of Estrogens in Cow Manure (mg/kg)				
	E3	BPA	DES	E2	EE2
0	97.40 ± 3.55	96.54 ± 0.42	100.22 ± 0.35	95.01 ± 1.01	72.49 ± 1.55
1.11	52.43 ± 1.60	1.21 ± 0.04	3.83 ± 0.13	9.96 ± 0.23	42.41 ± 2.76
1.57	43.28 ± 0.68	0.98 ± 0.03	2.81 ± 0.15	4.22 ± 0.07	26.54 ± 1.29
2.07	34.39 ± 2.16	0.58 ± 0.01	1.62 ± 0.18	3.06 ± 0.34	15.24 ± 1.81
2.56	15.92 ± 1.43	0.29 ± 0.03	0.48 ± 0.01	2.14 ± 0.44	12.11 ± 1.77
3.07	21.39 ± 3.77	0.57 ± 0.12	1.83 ± 0.08	3.03 ± 0.08	13.34 ± 1.55
3.59	29.35 ± 4.16	0.70 ± 0.04	2.36 ± 0.13	3.22 ± 0.21	14.60 ± 0.80

**Table 3:** Effect of the Fe(II) to H_2_O_2_ molar ratio on the residual concentrations of estrogens in cow manure by the Fenton oxidation process. Note: the H_2_O_2_ dosage was 2.56 mmol/g; the solid to water mass ratio was 2 g/mL; the initial pH value was 3.0; the reaction time was 24 h.

Fe(II) to H_2_O_2_ Molar Ratio/(M/M)	The Residual Concentrations of Estrogens in Cow Manure (mg/kg)				
	E3	BPA	DES	E2	EE2
0	97.40 ± 3.55	96.54 ± 0.42	100.22 ± 0.35	95.01 ± 1.01	72.49 ± 1.55
0.04	47.63 ± 1.41	1.46 ± 0.29	3.60 ± 0.29	3.42 ± 0.09	26.83 ± 1.59
0.05	32.95 ± 1.46	0.94 ± 0.09	3.21 ± 0.71	3.25 ± 0.42	21.00 ± 0.71
0.067	31.37 ± 3.44	0.67 ± 0.09	2.01 ± 0.37	2.21 ± 0.28	13.81 ± 1.01
0.10	18.89 ± 4.84	0.33 ± 0.01	0.75 ± 0.44	1.72 ± 0.19	11.95 ± 0.31
0.125	16.82 ± 1.32	0.32 ± 0.02	0.48 ± 0.48	0.98 ± 0.40	9.92 ± 1.25

**Table 4:** Effect of the solid to water mass ratio on the residual concentrations of estrogens in cow manure by the Fenton oxidation process. Note: the Fe(II) to H_2_O_2_ molar ratio was 0.10 M/M; the H_2_O_2_ dosage was 2.56 mmol/g; the initial pH values was 3.0; the reaction time was 24 h.

Solid to Water Mass Ratio/(g/mL)	The Residual Concentrations of Estrogens in Cow Manure (mg/kg)				
	E3	BPA	DES	E2	EE2
2	19.56 ± 5.16	0.38 ± 0.11	0.54 ± 0.07	0.81 ± 0.14	11.12 ± 1.13
1	46.08 ± 2.09	0.77 ± 0.17	1.44 ± 0.19	2.85 ± 0.34	17.68 ± 0.90
0.5	61.28 ± 1.17	1.26 ± 0.08	2.47 ± 0.13	3.34 ± 0.59	20.47 ± 1.04
0.33	65.66 ± 4.28	2.05 ± 0.14	3.58 ± 0.11	4.54 ± 0.38	41.84 ± 4.78

**Table 5:** Effect of initial pH on the residual concentrations of estrogens in cow manure by the Fenton oxidation process. Note: the Fe(II) to H_2_O_2_ molar ratio was 0.10 M/M; the H_2_O_2_ dosage was 2.56 mmol/g; the solid to water mass ratio was 2 g/mL; the reaction time was 24 h.

Initial pH Value	The Residual Concentrations of Estrogens in Cow Manure (mg/kg)				
	E3	BPA	DES	E2	EE2
3.0	15.57 ± 0.69	0.23 ± 0.09	0.62 ± 0.18	1.21 ± 0.72	11.51 ± 0.65
5.0	26.95 ± 0.90	0.54 ± 0.15	3.28 ± 0.51	1.72 ± 0.14	14.40 ± 1.14
7.0	38.56 ± 5.56	0.95 ± 0.11	4.69 ± 0.55	4.30 ± 0.15	15.71 ± 0.84
9.0	51.88 ± 7.22	1.22 ± 0.08	6.86 ± 0.22	6.65 ± 0.65	19.56 ± 0.57
11.0	61.74 ± 1.18	1.42 ± 0.04	8.78 ± 0.13	9.45 ± 0.30	22.90 ± 2.37

**Table 6:** Effect of reaction time on the residual concentrations of estrogens in cow manure. Note: Fe(II) to H_2_O_2_ molar ratio = 0.10 M/M; H_2_O_2_ dosage = 2.56 mmol/g; solid to water mass ratio = 2 g/mL; initial pH values = 3.0.

Reaction Time/h	The Residual Concentrations of Estrogens in Cow Manure/(mg/kg)				
	E3	BPA	DES	E2	EE2
6	25.94 ± 0.52	1.16 ± 0.20	3.47 ± 0.31	6.21 ± 0.87	23.52 ± 1.57
12	22.18 ± 1.69	0.51 ± 0.19	2.53 ± 0.33	4.18 ± 0.87	15.38 ± 0.64
24	14.62 ± 4.59	0.11 ± 0.04	0.80 ± 0.40	1.95 ± 0.50	11.71 ± 2.32
36	16.09 ± 2.13	0.15 ± 0.04	0.75 ± 0.13	1.79 ± 0.08	9.59 ± 1.71
48	15.72 ± 1.61	0.08 ± 0.06	0.66 ± 0.25	1.70 ± 0.11	11.56 ± 4.30
72	14.76 ± 1.35	0.02 ± 0.00	0.70 ± 0.58	1.52 ± 0.69	10.36 ± 0.97
